# Exploring the potential of microbial inoculant to enhance common bean (*Phaseolus vulgaris* L:) yield via increased root nodulation and soil macro-nutrients

**DOI:** 10.1371/journal.pone.0323854

**Published:** 2025-10-30

**Authors:** Sien Tigah Fortu, Aaron Suh Tening, Solange Takwi Ndzeshala, Christopher Ngosong

**Affiliations:** 1 Rhizobiology Group, Department of Agronomic and Applied Molecular Sciences, Faculty of Agriculture and Veterinary Medicine, University of Buea, Buea, South West Region, Cameroon; 2 Department of Agronomic and Applied Molecular Sciences, Faculty of Agriculture and Veterinary Medicine, University of Buea, Buea, South West Region, Cameroon; Universidade de Coimbra, PORTUGAL

## Abstract

Soil fertility constraints for common bean (*Phaseolus vulgaris* L.) production are often resolved using chemical fertilizers, but microbial inoculants can be used as a sustainable option/complement. Microbial inoculant formulation was tested on common bean in a randomized-complete-block-design field experiment with eight treatments and four replicates. The treatments included no input(control), chemical fertilizer(NPK), poultry manure(PM), Microbial inoculants(MI), NPK + PM, NPK + MI, PM + MI, and NPK + PM + MI. Microbial inoculant treatment had the highest fertilizer replacement value (proportion of chemical fertilizer that an alternative input can substitute) relative to poultry manure(41.6%) and chemical fertilizer(26.7%). The highest bean yield(3.46 tons ha^-1^) that was observed in the NPK + PM + MI treatment was significantly (*P* < 0.001) different from the microbial inoculant treatment(1.71 tons ha^-1^) and the control(1.42 tons ha^-1^). The highest 1000-grain weight (494 g) that occurred in NPK + PM + MI inoculant treatment was significantly (*P* < 0.05) different from the MI(457 g), NPK(448 g), PM(436 g), and control(421 g) treatments. Effective root nodules correlated positively (*P* < 0.05) with bean yield (*r* = 0.77) and 1000-grain weight (*r* = 0.90). Application of NPK + PM + MI had higher nodule number (9) and effectiveness than the control (2). Soil pH (6.6) was significantly (*P* < 0.05) higher in NPK + PM + MI than MI + PM (6.5), control (5.5) and NPK (5.0). Soil nitrogen and available phosphorus increased significantly (*P* < 0.05) in NPK + PM + MI 0.25% and 8.68 mg/kg, respectively, than the control(0.13% and 6.31 mg/kg), while higher potassium(2.80 cmol/kg) occurred in NPK than control(1.70 cmol/kg). These findings highlight the potential to explore the ability of bio-inoculant to boost root nodulation, enhance soil macro-nutrients, and modulate soil pH in bean production systems.

## 1. Introduction

Ensuring global food/nutrition security requires production of nutrient-rich foods such as common bean (*Phaseolus vulgaris* L.), which is a vital source of dietary protein [1,2]. Common bean is widely cultivated for subsistence and commercial purposes, and its production serves a dual role as soil fertility enhancer and food source to over 300 million people [3–5]. Global bean production was estimated at 28.5 million tons in 2023, and Africa contributed 29.21% (8.32 million tons) [6]. The global average yield of common bean is 0.75 tons ha ⁻ ¹, with Africa averaging about 0.78 tons ha ⁻ ¹. Cameroon produced only 390,098 tons at an average yield of 1.28 tons ha ⁻ ¹ compared to Mali with an exceptionally high yield of 10 tons ha ⁻ ¹ [6]. These high yields are achieved through improved crop varieties, intensive field management and use of inputs, and favourable agroecological conditions [7,8]. The current low crop yield in SSA is partly due to macro-nutrient deficiency (nitrogen–N, phosphorus–P, and potassium–K), coupled with pest/disease constraints [9,10].

Various management strategies are employed to grapple with biotic and abiotic challenges in crop production including chemical and organic inputs [3,11,12]. However, indiscriminate use of agro-chemicals threatens the sustainability of agricultural systems due to soil degradation, nutrient imbalance, salinization, and acidification, coupled with pest/disease effects [13,14]. Alternatively, beneficial microorganisms such as bacteria and fungi can improve soil nutrient availability via N-fixation and P-solubilization, mitigation of pest/disease effects, tolerance to biotic/abiotic stressors, boost nutrient uptake and crop performance [15,16]. Hence, microbial inoculation is being encouraged as a sustainable option to improve the yield of common bean [17], soybean [18] and maize [19], control banana mealybug pest [20], and improve soil physico-chemical properties [21]. Crop yield is largely determined by soil physico-chemical and biological properties, which can be modulated by unsustainable management practices that can jeopardize soil productivity [22–24].

Current calls to harness the benefits of the plant microbiome in agriculture are supported by recent field studies that demonstrated positive effects [20,21,25,26]. Microbial inoculants may comprise single strains or combinations of symbiotic and non-symbiotic bacteria or fungi with the ability to fix atmospheric nitrogen (*Rhizobium*), solubilize soil phosphate (*Bacillus, Pseudomonas*). These microbes enhance nutrient uptake, improve root architecture, induce stress resistance, and suppress crop pests/pathogens [16,27]. Microbes can mediate important soil functions such as N fixation, P or K solubilisation and mineralisation, phytohormones and siderophores production [28–32]. They promote urease activity in the soil that supplies N to plants by hydrolysing nitrogenous compounds and boost the nitrogenase enzyme complex that fixes atmospheric nitrogen [21,33]. Plants and microorganisms release phosphatase enzymes that facilitate mobilization of minerally or organically bound phosphates into bioavailable forms and enhance P cycling [34,35]. Microbes also produce siderophore that facilitates solubilization of iron from rhizosphere minerals or organic compounds by binding and reducing Fe^3+^ to Fe^2+^ [32]. Hence, microbial inoculants play vital roles in plant-soil relations that are often ignored, but they contribute significantly to crop performances.

Nonetheless, despite the observed positive effects of beneficial microbes, their availability in local markets and adoption is relatively low. Hence, this study aims to evaluate the potential of locally produced microbial inoculant consortium to effectively improve common bean grain yield by enhancing root nodulation and soil macro-nutrient contents. It was hypothesized that microbial inoculant would increase common bean grain yield via enhanced root nodulation, modulation of soil pH, and enhancement of soil macro-nutrient contents as compared to the chemical or organic amendments and the control. The objectives of this study were to: (1) Assess the effect of a locally produced microbial inoculant consortium on common bean yield and root nodulation; and (2) Evaluate the impact and effectiveness of locally produced microbial inoculant consortium on soil pH and macro-nutrient status relative to chemical and organic amendments.

## 2. Materials and methods

### 2.1. Experimental site

This experiment was conducted in 2021 at the Teaching and Research Farm of the Faculty of Agriculture and Veterinary Medicine, University of Buea, Cameroon, between May and August. The site is located at the foot of Mount Cameroon (4100 m) in the South West Region, at about 1000 m above sea level, with coordinates of latitude 04^0^ 8’ 55.1’‘ N and longitude 09^0^16’ 53’‘ E. Buea has a mono-modal rainfall pattern with rainy season from March to October and 2,085 mm annual average on the leeward side of the mountain and 9,086 mm on the windward side. The dry season runs from November to February, with mean monthly temperatures ranging from 19–30 °C and 28 °C annual mean, with decreasing soil temperature from 25 °C to 15 °C at 10 cm depth as elevation increases from 200–2200 m above sea level [36,37]. The relative humidity ranges between 85–90%, with annual sunshine ranging from 900–1200 hours. The soil has a pH of 6.60 with a texture made up of sand (68%), silt (18%), and clay (14%).

### 2.2. Experimental setup

The field experiment was conducted from May to August 2021 on a land that was previously cultivated with maize (*Zea mays*), cowpea (*Vigna unguiculata*), groundnuts (*Arachis hypogaea*), African nightshade (*Solanum nigrum*) and amaranth (*Amaranthus* spp.). The land was cleared manually using a cutlass and demarcated into 32 plots (4 m × 4 m). The experiment was set up as a randomised complete block design (RCBD) with eight treatments and four replicates. The treatments included no input (control), chemical fertilizer (NPK), poultry manure (PM), microbial inoculants (MI), NPK + PM, NPK + MI, PM + MI, and NPK + PM + MI. Experimental units were separated by 1-m gaps and a 2-m buffer zone surrounded the experimental site. In addition to baseline analysis of a pre-planting composite field soil sample that was collected from 0–15 cm depth, a sample of poultry manure was also analysed. (**Table 1**).

**Table 1:** Chemical composition of poultry manure and baseline soil properties of the experimental field.

Parameters	pH(H_2_O)	Tot. Nitrogen (%)	Av. Phosphorus (mg/kg)	Potassium	Calcium cmol/kg	Magnesium
Soil	6.60 ± 0.18	0.25 ± 0.02	8.02 ± 0.23	1.62 ± 0.21	2.96 ± 0.30	1.24 ± 0.14
Poultry manure	7.8 ± 0.01	3.84 ± 0.01	8.35 ± 0.11	3.18 ± 0.01	5.2 ± 0.01	1.37 ± 0.01

### 2.3. Preparation of microbial inoculum

A bio-inoculant consortium of beneficial bacteria (**Table 2**) was formulated, comprising symbiotic *Bradyrhizobium japonicum* and *Kosakosania radicincitans,* and non-symbiotic *Arthrobacter* spp. (03), *Bacillus* spp. (03), *Lysinibacillus* sp. (01), *Paenibacillus* spp. (02), and *Sinomonas* sp. (01). Serial dilution, plating on nutrient media (Standard nutrient agar I, Carl Roth, Germany), incubating, and counting of colony-forming units (CFUs) were done to check bacterial inoculum viability before seed inoculation. *B. japonicum* was obtained from the Soil Microbiology Laboratory of the Biotechnology Center, University of Yaoundé I, Cameroon. *K. radicincitans* was isolated from the phyllosphere of winter wheat in Germany, deposited in NCBI as DSM 16656T GenBank: CP018016.1, CP018017.1, CP018018.1, and stored at the Rhizobiology Laboratory (RhizoLab), Faculty of Agriculture and Veterinary Medicine, University of Buea, Cameroon [38,39]. The non-symbiotic bacteria were isolated from the rhizosphere of maize plants in Cameroon [27] and stored at the Rhizobiology Laboratory. The selected strains were included for their known functions in N-fixation, P-solubilization, and growth hormone production [27,29].

**Table 2:** Bacterial strains used in the formulation of microbial inoculant and their potential functions in the inoculant consortium [27,29].

Microbial isolates	Genus	Origin	Reason for selection
Nka11 and Nks5	*Bradyrhizobium*	Cameroon – Cowpea Rhizosphere	N-fixation
V64, V84 & V127	*Arthrobacter*	Cameroon – Maize Rhizosphere	Siderophore production and P-solubilization
VA9, V22 & V65	*Bacillus*		
V47	*Lysinibacillus*		N-fixation
V12 & V18	*Paenibacillus*		N-fixation and P-solubilization
V4	*Sinomonas*		Siderophore production and P-solubilization
D5/23	*Kosakonia*	Wheat leaf, Germany	N-fixation, Siderophore production and P-solubilization

A colony of *B. japonicum* was collected from the inoculum stock and transferred into a 500 mL flask containing 100 mL sterilized yeast mannitol broth (YMB), and incubated in a shaker at 28 °C at 200 rpm for 48 hrs. From an individual stock culture of the other bacteria, a pure colony was collected and transferred into a 500 mL flask containing 100 mL sterilized nutrient broth (Standard nutrient broth I, Carl Roth, Germany), and incubated at 28 °C for 24–48 h. The individually cultured bacteria were assembled into a microbial consortium in a 5 L container. 20.5g of sugar was added to serve as an adjuvant given that *B. japonicum* is not sticky. Common bean seeds were immersed in the microbial inoculant consortium (e.g., 1 kg seeds per 100 mL of bio-inoculant) and thoroughly mixed. Seeds were removed from the inoculum and air-dried for 1 hr before planting [40]. All inoculation procedures were performed under shade to maintain the viability of the microbes.

### 2.4. Soil fertility amendments

The NPK fertilizer (20:10:10 + CaO; ADER^®^ Cameroon) was bought from a local market in Buea. Poultry manure (obtained from a 2-month-old substrate) was collected from a certified poultry farm in Buea and sun-dried for two weeks before application to reduce moisture content, suppress pathogenic organisms, stabilize nutrient composition, and prevent phytotoxic effects associated with the use of fresh manure. Granular NPK fertilizer was applied manually by ringing at about 5 cm from plants, as a split dose of 5 g NPK per stand at 2 and 4 weeks after sowing [12]. Poultry manure was applied two weeks before planting at 20 tons ha^-1^ [41].

### 2.5. Crop cultivation and management

Untreated bean seeds (ECA PAN 021) were purchased from a local market and sown manually at 35 × 35 cm spacing. Two seeds were sown per stand at about 3–4 cm depth and later thinned to one vigorous plant per stand after germination, giving a total of 130 plants per plot, equivalent to 82,000 plants per hectare. Two weeks before planting, the field was sprayed with pre-emergence systemic herbicide (METROPOLE^®^ - Active ingredient Metribuzin) at 15 mL per 16 L of water. After planting, plots were monitored regularly for weed emergence and weeded manually. The entire crop cycle depended on the local rainfall regime and was regularly monitored for pest infestation and disease incidence throughout the crop growth.

### 2.6. Data collection

#### 2.6.1. Common bean growth and yield parameters.

Ten randomly selected plants from the middle rows in each plot were tagged for growth and yield data collection. Plant growth components (plant height, stem girth, leaf area, number of branches, and number of leaves) were collected beginning two weeks after germination, and then every week for 6 weeks. Stem girth expressed in centimetres (cm) was measured using a graduated tape at the plant base above the soil surface. Bean yield components such as number of flowers, number of pods, length of pods, number of seeds per pod were collected before harvest, and fresh and dry weight of pods and seeds were collected at harvest. All measurements were collected at the same phenological stage (physiological maturity) for consistency. Grains were harvested and oven-dried at a constant temperature of 60 °C for three days [42]. The 1000-grain weight (g) was calculated by taking the weight of randomly selected 1000 grains from each treatment replicate and weighing them. Grain yield was calculated using the following formula [43]:


$$\text{ Grain yield }(kg/ha)=\frac{\text{Plot yield }(\text{kg})\text{ x 10},\text{000 m2}}{\text{Plot size in m2}}\times \;\text{1}\text{0}\text{0}$$ (1)

Additionally, fertilizer replacement value (FRV) was calculated as a useful parameter to evaluate the effectiveness of alternative amendments, e.g., comparing microbial inoculant with chemical or organic fertilizers and their combinations. The FRV was calculated based on grain yield using the formula below [44,45]:


$$\text{ FRV }(\%)=\frac{\text{Grain yield }(\text{Pv})-\text{Grain yield }(\text{Po})}{\text{Grain yield }(\text{Pi})-\text{Grain yield }(\text{Po})}\times \;\text{1}\text{0}\text{0}$$ (2)

Where Pv refers to microbial inoculant, Po is the control, and Pi is chemical or organic fertilizers and their combinations.

#### 2.6.2. Soil chemical properties.

Post-planting (at physiological maturity) soil samples were collected and analyzed for routine parameters (pH, total N, available P, organic carbon, exchangeable bases and particle size distribution) at the Environmental and Analytical Chemistry Laboratory, University of Dschang, Cameroon. Soil samples were collected at 0–15 cm-depth using an auger and stored in polythene bags. Soil and manure samples were air-dried on plastic trays at room temperature, ground, and passed through a 2-mm sieve. Soil pH was determined at 1:2.5 soil/water suspensions using a digital pH meter. The instrument was calibrated daily with buffer solutions at pH 4.00, 7.00, and 10.00. The detection range of the instrument was 0.00–14.00 pH units, with a resolution of ±0.01 and a detection limit of ±0.02 pH units. Soil available phosphorus was determined by the Bray II method at ~0.05–0.10 mg/kg detection limit [46], organic carbon (OC) by the Walkley and Black wet digestion method at ~0.05% OC detection limit [47], and total nitrogen by the Kjeldahl digestion method at ~0.005% N (50 ppm) detection limit [48]. Soil exchangeable bases were extracted with 1 N ammonium acetate (NH_4_OAc) solution at pH 7. After extraction, calcium and magnesium were determined by complexometric titration, while sodium and potassium were determined using a flame photometer [49]. Particle size distribution was determined by the pipette method and textural class was assigned according to the USDA textural triangle [46].

#### 2.6.3. Root nodulation.

**Fig 1:**
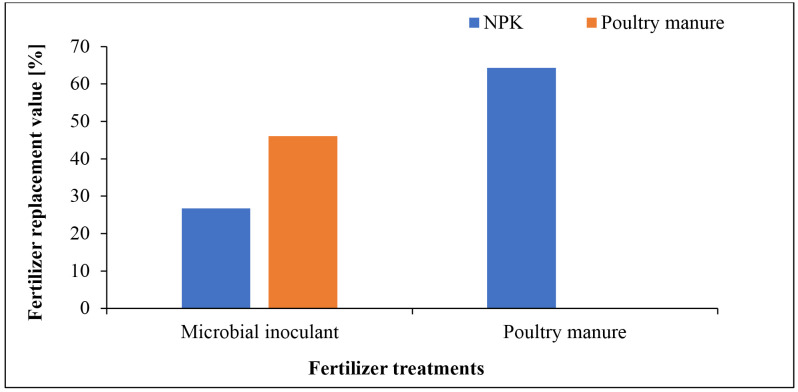
Fertilizer replacement value of microbial inoculant treatment relative to NPK and poultry manure fertilizers, and poultry manure relative to NPK fertilizer.

Root nodulation was assessed 65 days after sowing (late flowering), where ten randomly selected plants per treatment plot were uprooted and placed in a water-filled basin. Roots comprising nodules were gently washed with tap water and filtered through a 250-mm sieve. Total number of nodules was counted and all the washed nodules were dissected using a razor blade to determine the number of effective nodules based on the pigmentation S1 Fig 1. Nodules with pink or red colouration were recorded as effective in N-fixation due to leghemoglobin oxygen carrier essential for nitrogen fixation, and white nodules were considered as non-effective [50].

### 2.7. Data analysis

All analyses were done using SPSS (Ver. 23) statistical software package, while Microsoft Excel (2019) was used to process graphs and tables. Normality of data was checked using Kolmogorov–Smirnov test, which determines whether a variable’s distribution significantly deviates from a normal distribution. Levene’s test (tests equality of error variances) was used to confirm homogeneity of variances between treatment groups. Data for selected soil physico-chemical properties, root nodulation, growth, and yield parameters were subjected to one-way analysis of variance (ANOVA) to test effects of treatments. Significant means were separated using Tukey’s Honesty Significant Difference Test (Tukey’s HSD, *P* < 0.05). Pearson Correlation (*P* < 0.05) was performed to determine the degree of association between variables. Effect sizes (η²) were also reported where applicable to determine the proportion of total variance in the measured parameter relative to treatment effects [51].

## 3. Results

### 3.1. Fertilizer replacement value and bean performance

**Fig 2:**
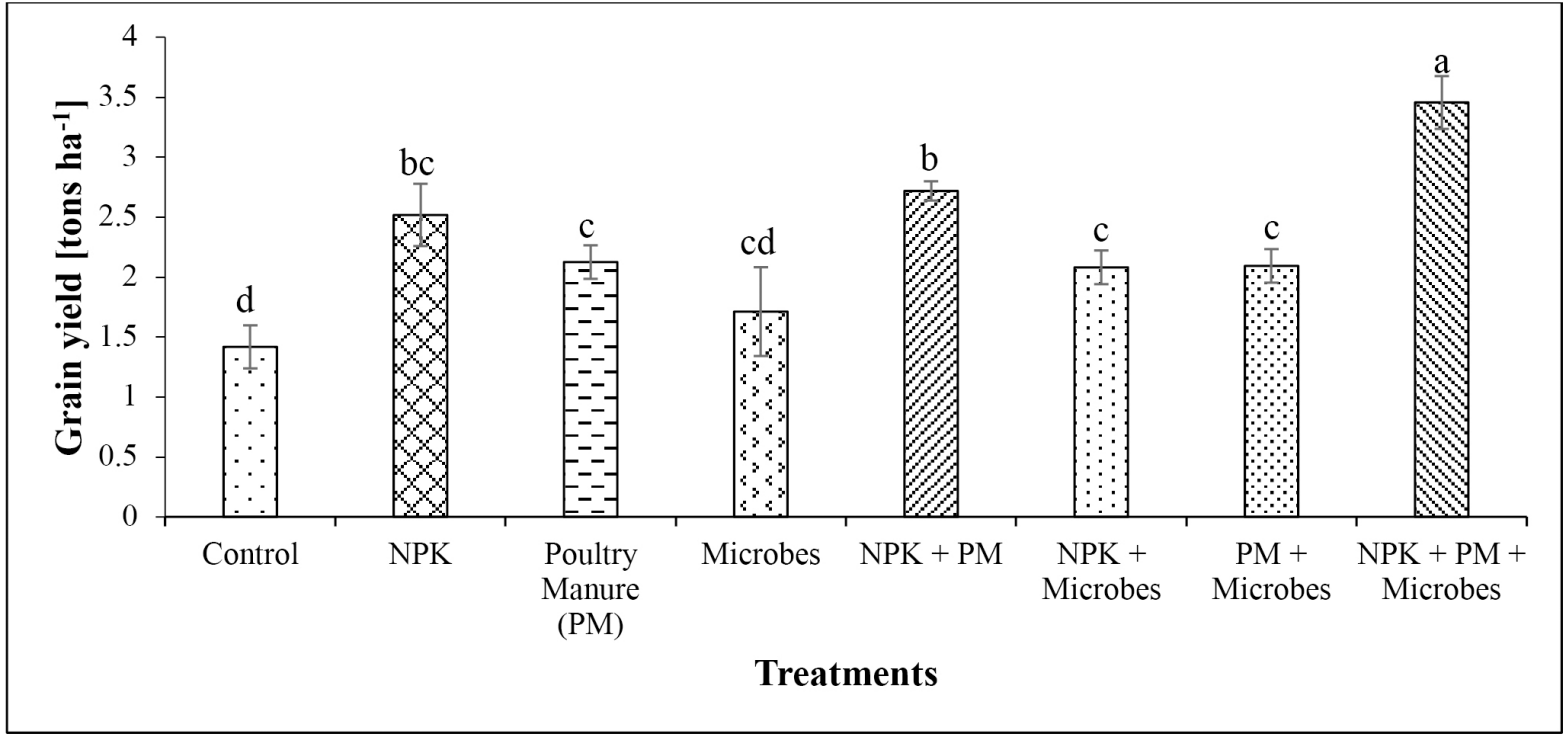
Impact of treatments on common bean grain yield (tons ha^-1^, Mean  ± SD, *n* = 4). Values with different letters are significantly different (Tukey’s HSD, *P* < 0.05).

**Fig 3:**
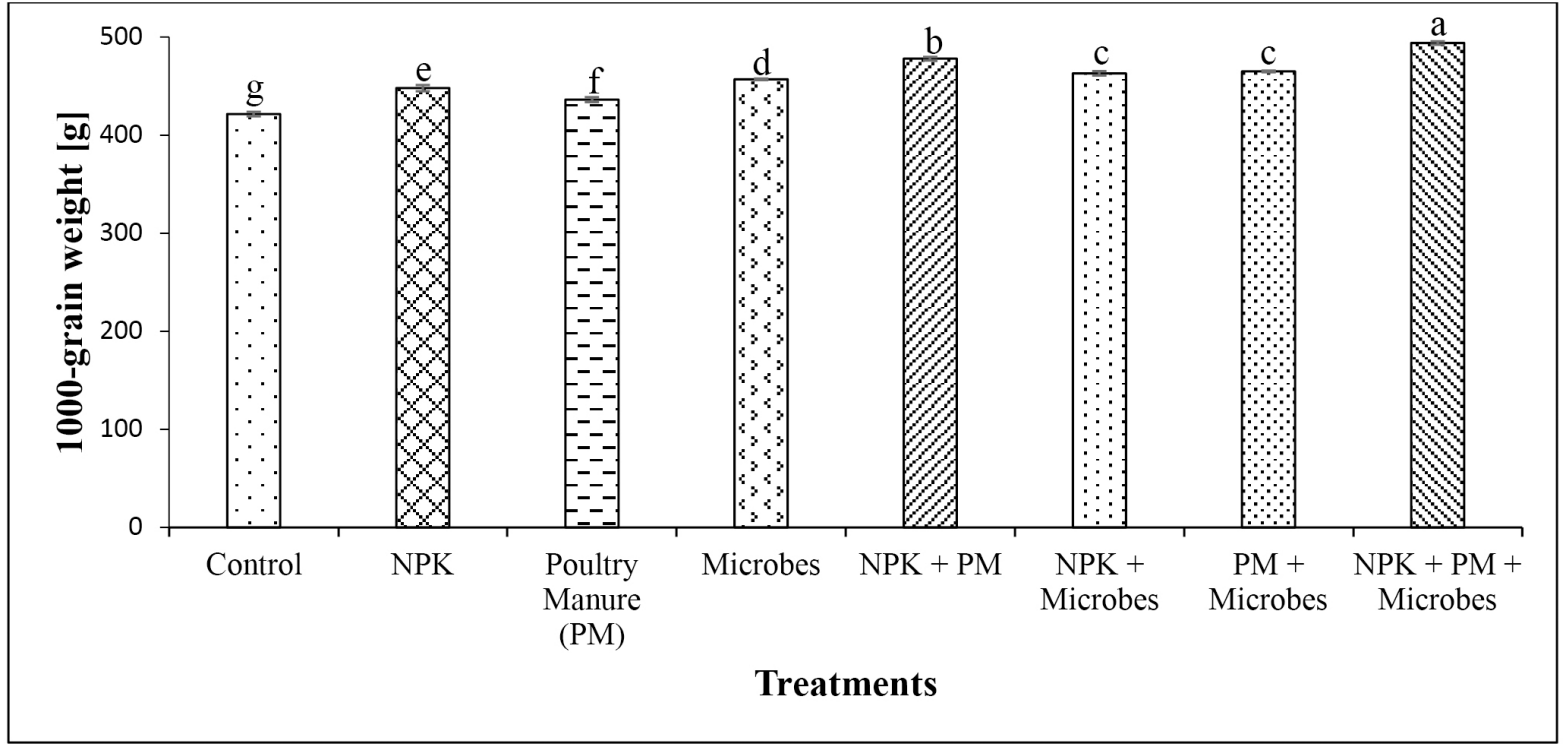
Impact of treatments on 1000-grain weight of common bean (Mean  ± SD, *n* = 4). Values with different letters are significantly different (Tukey’s HSD, *P* < 0.05).

All parameters analysed were higher in poultry manure over baseline soil (**Table 1**). Poultry manure had higher NPK (3.84% N, 8.35 mg/kg P and 3.18 cmol/kg K) compared to the baseline soil (0.25% N, 8.02 mg/kg P and 1.62 cmol/kg K). Microbial inoculants had higher fertilizer replacement value (i.e., the proportion of chemical fertilizer that can be substituted by an alternative input based on crop yield response) relative to poultry manure (41.6%) and NPK (26.7%), while poultry manure had 64.3% higher fertilizer replacement value relative to NPK (**Fig 1**). Common bean yield ranged between 1.42 and 3.46 tons ha^-1^, translating to 21% yield increase with microbial inoculant and 143% increase for the NPK + PM+Microbes treatment compared to the control. This increased significantly (*P* < 0.001, **Fig 2**) in all treatments relative to the control. The weight of 1000 bean grains ranged from 421–494 g and increased significantly (*P* < 0.001, **Fig 3**) in all treatments compared to the control (421 g), with the highest in the integrated application of chemical, organic and microbes (494 g). Bean yield (*r *= 0.77) and 1000-grain weight (*r* = 0.90) correlated significantly (*P* < 0.05) with the number of effective nodules. Integrated application of chemical, organic and microbes resulted in significantly (*P* < 0.05, **Table 3**) higher plant height (41.7 cm) compared to the other treatments, with the lowest in the control (29.1 cm). A similar trend was observed for the number of leaves (*P* < 0.05, **Table 3**), with the highest in integrated application of chemical, organic and microbes (15) and the lowest in the control (11). There was no significant difference between treatments on the stem girth, leaf area, and number of branches.

**Table 3:** Impact of treatments (Mean ± SD, *n* = 4) on plant growth parameters.

Treatments	Plant height (cm)	Number of leaves	Stem girth (cm)	Leaf area (cm^2^)	Number of branches
Control	29.1 ± 4.1^c^	11 ± 2^ab^	2.0 ± 0.3^a^	78.8 ± 19.9^b^	5 ± 1.0^a^
NPK	36.0 ± 2.5^abc^	13 ± 2^ab^	2.1 ± 0.4^a^	99.7 ± 21.3^ab^	6 ± 1.4^a^
PM	38.5 ± 3.1^ab^	13 ± 1^ab^	2.3 ± 0.2^a^	106.6 ± 10.3^ab^	6 ± 1.1^a^
MI	33.7 ± 1.3^bc^	11 ± 1^b^	2.2 ± 0.1^a^	91.8 ± 12.4^ab^	5 ± 0.5^a^
NPK + PM	37.0 ± 3.1^ab^	12 ± 2^ab^	2.5 ± 0.4^a^	115.8 ± 35.5^ab^	7 ± 0.5^a^
NPK + MI	33.8 ± 2.7^bc^	11 ± 2^ab^	2.0 ± 0.3^a^	85.6 ± 27.5^ab^	5 ± 0.6^a^
PM + MI	38.9 ± 4.8^ab^	11 ± 1^b^	2.2 ± 0.4^a^	92.7 ± 19.5^ab^	6 ± 0.8^a^
NPK + PM + MI	41.7 ± 3.8^a^	15 ± 2^a^	2.3 ± 0.1^a^	117.7 ± 10.6^a^	6 ± 0.4^a^

Values within a column with different letter(s) are significantly different (Tukey’s HSD, *P *< 0.05).

Control = no input; NPK = nitrogen, phosphorus and potassium; PM = poultry manure; MI = microbes

### 3.2. Soil chemical properties

Treatments significantly (*P* < 0.05) affected the post-planting soil (**Table 4**). Soil pH differed significantly (*P* = 0.00000134, η²: 0.9708) across treatments, with the highest in the integrated application of chemical, organic and microbes (6.53), followed by chemical and microbe (6.43), and the lowest in chemical input (5.0). Total soil N differed significantly (*P* = 0.003340, η²: 0.6907) across treatments with the highest in the integrated application of chemical, organic and microbes (0.25%) and the lowest in the control (0.13%). Soil available phosphorus also varied significantly (*P* = 0.0348, η²: 0.5631) across treatments, with the highest in the integrated application of chemical, organic and microbes (8.68 mg/kg), and the lowest in the control (6.31 mg/kg). Sole application of NPK recorded the highest (*P* = 0.0085, η²: 0.6463) potassium (2.8 cmol/kg) compared to the (1.7 cmol/kg).

**Table 4:** Effect of treatments (Mean ± SD, *n* = 4) on soil physico-chemical properties at physiological maturity of common bean.

Treatments	pH (H_2_O)	N (%)	Av. phosphorus (mg/kg)	Potassium	Calcium cmol/kg	Magnesium
Control	5.50 ± 0.17^b^	0.13 ± 0.01^c^	6.31 ± 0.16^d^	1.70 ± 0.22^b^	2.04 ± 0.54^c^	0.93 ± 0.12^c^
NPK	5.00 ± 0.17^c^	0.14 ± 0.03^bc^	7.04 ± 0.36^c^	2.80 ± 0.36^a^	3.92 ± 0.39^a^	1.19 ± 0.11^b^
PM	5.53 ± 0.06^b^	0.17 ± 0.04^bc^	6.72 ± 0.53^c^	2.30 ± 0.28^a^	2.37 ± 0.30^b^	1.64 ± 0.36^a^
MI	5.60 ± 0.01^b^	0.14 ± 0.02^bc^	7.70 ± 0.35^b^	2.59 ± 0.08^a^	2.52 ± 0.41^b^	1.83 ± 0.58^a^
NPK + PM	5.57 ± 0.15^b^	0.12 ± 0.04^bc^	6.41 ± 0.17^d^	2.79 ± 0.38^a^	2.77 ± 0.51^a^	2.20 ± 0.28^a^
NPK + MI	6.43 ± 0.06^a^	0.16 ± 0.04^b^	8.51 ± 0.43^a^	2.36 ± 0.22^a^	2.27 ± 0.68^b^	1.65 ± 0.30^a^
PM + MI	6.53 ± 0.06^a^	0.22 ± 0.06^ab^	7.97 ± 0.27^b^	2.78 ± 0.21^a^	2.78 ± 0.33^b^	1.68 ± 0.24^a^
NPK + PM + MI	6.53 ± 0.12^a^	0.25 ± 0.01^a^	8.68 ± 0.42^a^	2.58 ± 0.38^a^	2.37 ± 0.30^b^	1.35 ± 0.12^b^

Values within a column with different letter(s) are significantly different (Tukey’s HSD, *P* < 0.05). Control = no input; NPK = nitrogen, phosphorus and potassium; PM = poultry manure; MI = microbes

### 3.3. Root nodulation

**Fig 4:**
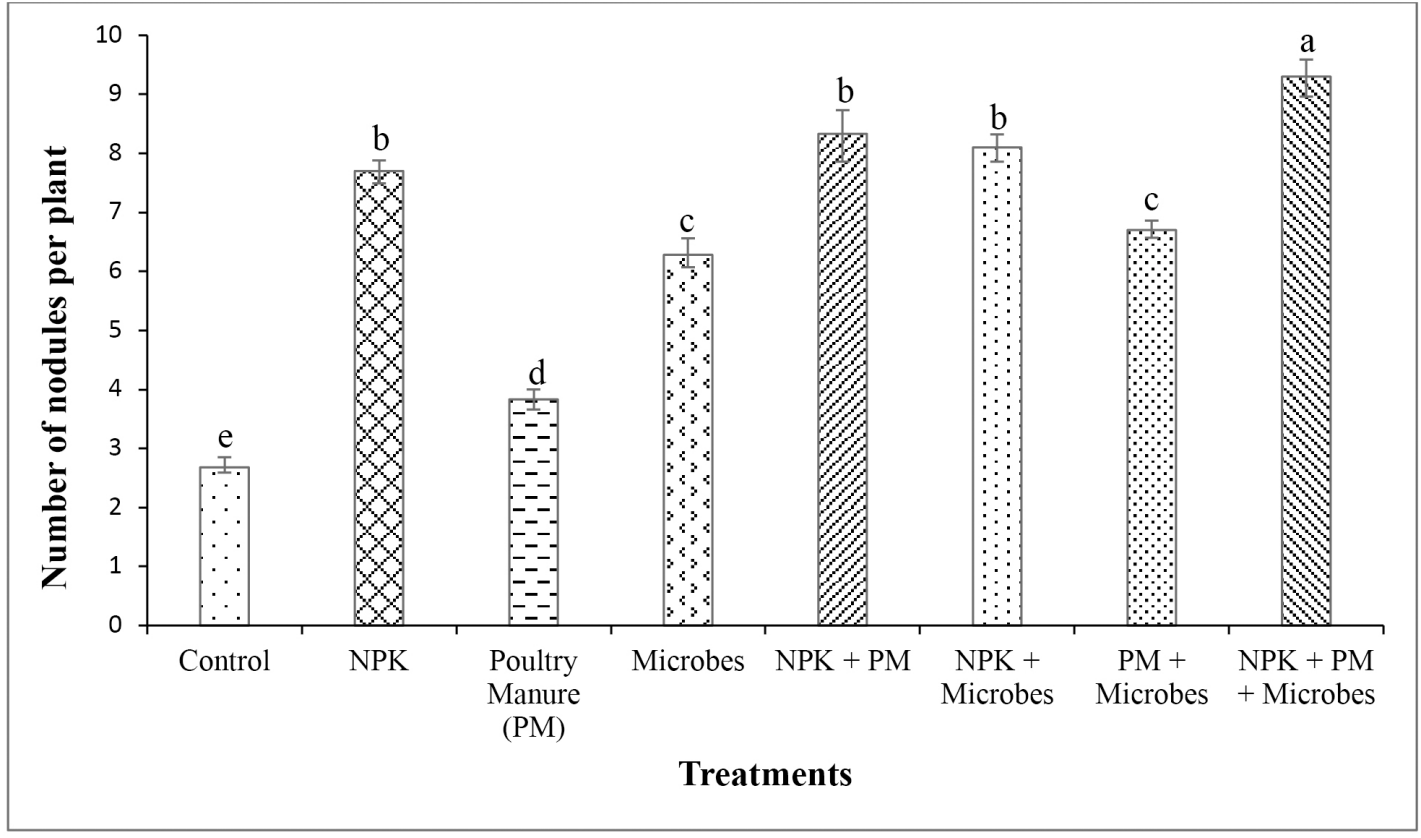
Influence of treatments on number of root nodules (Mean  ± SD, *n* = 4). Values with different letters are significantly different (Tukey’s HSD, *P* < 0.05).

**Fig 5:**
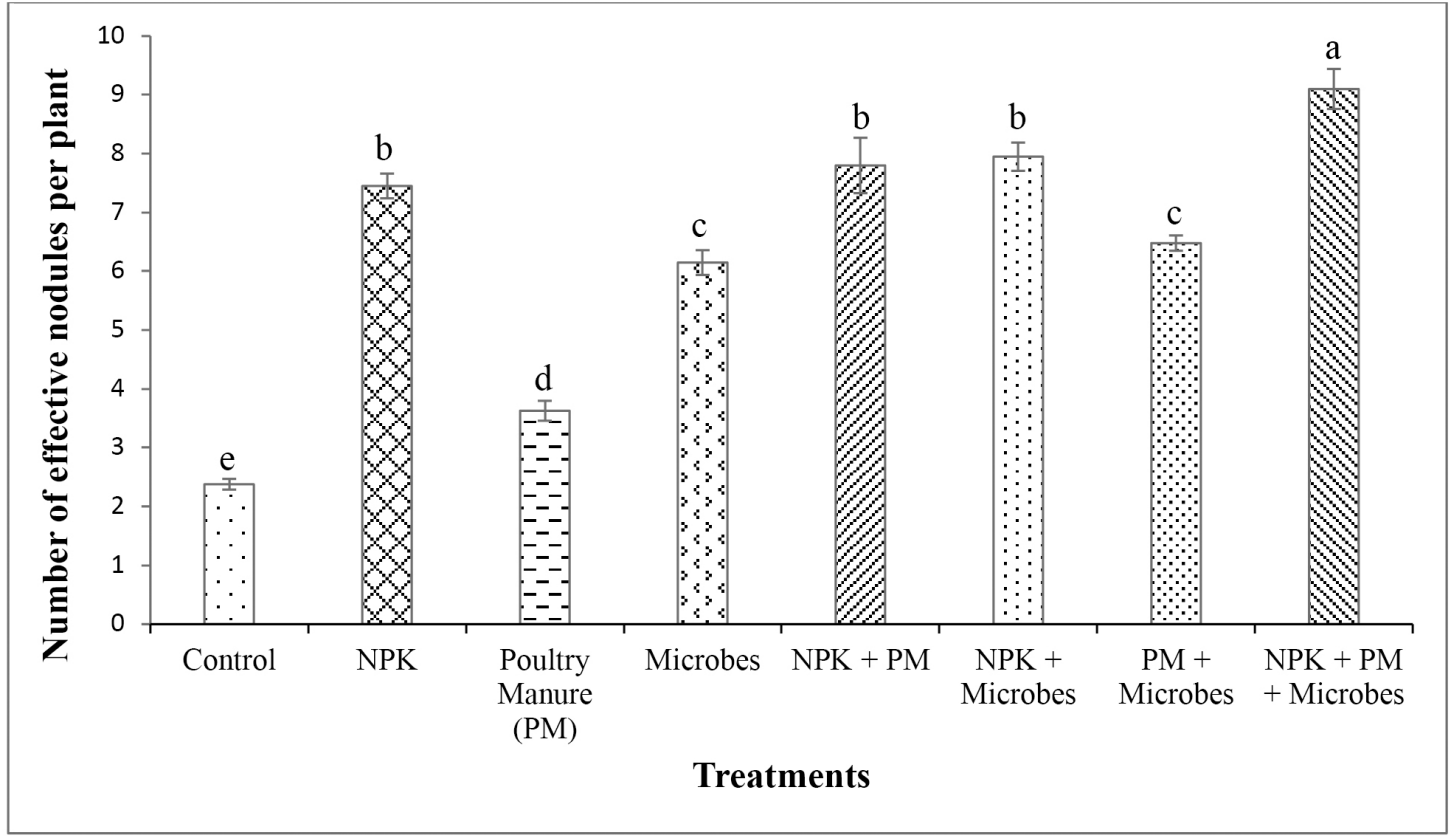
Influence of treatments on number of effective root nodules per plant (Mean  ± SD, *n* = 4). Values with different letters are significantly different (Tukey’s HSD, *P* < 0.05).

Treatments caused significant (*P* < 0.05) effects on bean root nodulation parameters (**Fig 4**), with the highest number of nodules per plant in the integrated application of chemical, organic and microbes (9), which differed significantly from the other treatments and the control (3) with the lowest (*P* < 0.001). Similarly, the highest (9) number of effective root nodules per plant was recorded in the integrated application of chemical, organic and microbes, and the lowest in the control (2). The number of nodules and number of effective nodules also differed significantly (*P* < 0.001, **Fig 5**), and increased in plants inoculated with microbes compared to control.

## 4. Discussion

### 4.1. Impact of treatments on common bean yield

The results obtained demonstrated that integrating microbial inoculants with organic and chemical fertilizers significantly improved common bean yield. The higher bean yield for NPK, poultry manure, microbial inoculant, and their integrated application compared to the control reflects constant nutrient supply during the crop life cycle that boosts productivity [12]. Microbial inoculants can establish themselves in the rhizosphere, facilitating better nutrient acquisition during crop growth, especially in legumes such as common bean, which relies on symbiotic relationships with N-fixing bacteria (*Rhizobium*) to meet their N requirements [52,53]. El-Azeem [54] reported that co-inoculating *Rhizobium* with *Pseudomonas fluorescens* and siderophore-producing PGPR resulted in enhanced nodulation, plant nutrient uptake, and substantial increase in common bean yield component such as pods per plant and seed weight, as compared to sole inoculation. Similarly, Huang *et al*. [55] found that co-inoculating *Bacillus* species with *Rhizobia* led to significant improvement in nodule weight and common bean biomass. Inoculating common bean varieties with *Rhizobium* increases grain yield by enhancing nodulation and improving nitrogen nutrition [8,56,57]. This aligns with Bojórquez *et al*. [58], who found that co-inoculating *Rhizobium* with PGPB led to improved yield and health of legumes compared to sole *Rhizobium* inoculation. In addition to improving plant nutrient uptake, microbial inoculants (e.g., *Kosakonia*, *Arthrobacter,* and *Bacillus*) can also enhance resilience to environmental stress, thereby, contributing to yield stability and reducing the need for synthetic fertilizers [17,59,60]. Furthermore, the presence of phosphorus solubilizers and siderophore-producing bacteria such as *Arthrobacter*, *Kosakonia,* and *Sinomonas* in the microbial inoculant consortium likely enhanced phosphorus availability to plants and iron uptake for chlorophyll synthesis and enzyme function [61–63], leading to increased crop performance especially under phosphorus limiting conditions [53,64]. This likely increased photosynthetic efficiency which resulted in improved crop growth and biomass accumulation [63,65]. Increased nutrient supply for the integrated application of chemical, organic and microbial inputs resulted in significant increase in nitrogen and phosphorus uptake by plants that eventually influenced common bean yield [66]. The increased symbiotic activity with more N-fixation in plants likely enhanced grain yield [29,39,67] as demonstrated by the significant positive correlation between nodule effectiveness and 1000-grain weight. Poor bean performance in the control treatment emphasizes the need for fertilizer inputs to boost soil fertility and productivity in the study area. In sum, the findings of this study are consistent with the hypothesis that inoculation with beneficial microbes will increase common bean yield via enhanced root nodulation and soil fertility status.

### 4.2. Effect of treatments on soil chemical properties

The enhanced soil fertility with treatment application highlights the role of organic and inorganic fertilizer amendments in improving soil fertility [68,69] and emphasizes the challenges of low soil fertility in cropping systems. The ability of treatments to buffer soil pH, with low values in NPK, highlights the contribution of NPK to soil acidification [70,71]. Under acidic pH, crop yield might be reduced as N use efficiency decreases, while excess reactive nitrogen could be lost, thereby generating H^+^ ions [72,73]. S2 Table 1 contains a summary table comparing pre- and post-treatment soil properties. The poultry manure used in this study had a high pH of 7.8 and high Ca^2+^(5.2%) and Mg^2+^(1.37%) contents, which could have a direct and rapid liming effect, proton exchange between soil and organic manure, which often contains some phenolic and humic materials [74,75]. The impacts of poultry manure and microbial applications in modulating the soil pH are consistent with Meza *et al*. [76] and Ogundijo *et al*. [74], who reported a significant increase in soil pH with the application of organic amendments. This buffering effect may be attributed to microbial secretion of organic acids and cation exchange processes that improve soil structure and nutrient retention [77]. PGPB such as *Bradyrhizobium* and *Kosakonia* improve crop yield via increased plant nutrient uptake and tolerance to stress, and their integration with poultry manure can optimize plant nutrient uptake, boost productivity and promote sustainability, as seen with the 41.6% fertilizer replacement value of microbial inoculants relative to poultry manure [76,78]. The observed high soil N level for the integrated application of chemical, organic and microbes may be due to high N content of the poultry manure, presence of N-fixing bacteria (*Bradyrhizobium*, *Kosakonia, Lysinibacillis* and *Paenibacillus*) in the microbial inoculant consortium and residual N from the chemical fertilizer input [56,74]. High P and K levels also observed in the integrated application of chemical, organic and microbe may be due to high K in poultry manure or *Bacillus* and *Paenibacillus* mediated K uptake by plants [79], residual K from chemical fertilizer input, and solubilization by *Arthrobacter*, *Bacillus*, *Kosakonia, Paenibacillus* and *Sinomonas* [56,80]. The integration of chemical, organic and microbes may lead to nutrient leaching if not well managed, which requires monitoring to avoid off-site impacts [81]. *Arthrobacter*, *Kosakonia,* and *Sinomonas* can enhance plant nutrient solubilization and uptake, improving the overall soil nutrient profile [54,64], which is important in low-fertility soils where common beans and other legumes are grown. This interaction of PGPB in soil not only boosts nitrogen levels but also enhances the availability of other essential nutrients such as P and iron, due to enhanced mineralization and microbial activity that contributes to better plant health and productivity [82–84]. These findings support the hypothesis of this study that beneficial microbes will modulate soil pH and enhance the soil fertility of common bean fields.

### 4.3. Influence of treatments on root nodulation

The observed number of root nodules is within the expected range for common bean under tropical agroecological conditions, indicating effective root colonization [85,86]. The increased number and effectiveness of root nodules in treatments inoculated with microbes is likely due to the influence of *Bradyrhizobium* on N-fixation, which is reflected in the soil N-content that likely resulted in greater photosynthate production for higher grain yield [56,87]. The integrated application of chemical, organic and microbes likely contributed to improved symbiotic N reflected in the high number of effective nodules, considering that common bean is a poor N-fixer due to delayed nodule formation with insufficient nodule mass, and ineffective nodules [50,88]. Samago *et al*. [56] and Milcheski *et al*. [89] demonstrated that inoculation with specific rhizobial strains significantly increased nitrogen availability, enhanced nodulation and overall biomass production, leading to higher grain yields. Moreover, common bean has high P-demand for optimum nodulation and growth, which boosts atmospheric N fixation [56,90]. In addition, P initiates nodule formation, increases nodule formation and functions [66,91]. P-solubilizing bacteria in the bio-inoculant consortium such as *Arthrobacter*, *Bacillus*, *Kosakonia, Paenibacillus* and *Sinomonas* likely increased the P-content of soil and enabled optimum soil N-P balance that fostered root nodulation and effectiveness [29,39,67]. The soil also plays a critical role in the effectiveness of microbial inoculants, as the presence of native rhizobia in soil can enhance their symbiotic relationship with common beans. Kawaka *et al*. [92] demonstrated that high populations of native rhizobia in certain soils contributed to effective root nodulation and N fixation. Additionally, P application in combination with *Bradyrhizobium* inoculation increased grain yield, as P availability is essential for N fixation [56,93]. The increase in the number of nodules and nodule effectiveness could be attributed to improved nutrient availability such as P and Ca, which might have been supplied by chemical and organic inputs [50,94]. Hence, the decrease in the number of nodules under poultry manure compared to chemical fertilizer is probably due to constant nutrient supply by poultry manure, which reduced plant dependence on N-fixation [12,86]. Also, interaction of native soil rhizobia with introduced microbial strains may enhance or inhibit nodulation depending on competitiveness [85,95]. These results support the hypothesis that microbial inoculants will enhance root nodulation and nitrogen fixation, thereby increasing common bean grain yield, and the impact may vary under different soil types or climates.

## 5. Conclusion

The locally produced microbial inoculant consortium significantly increased the yield of common bean as compared to the control, while the integrated treatments resulted in the highest yields. Thereby highlighting the importance of microbial inoculants and integrated soil fertility management strategies in increasing common bean yield. Additionally, microbial inoculant consortium and all other treatments increased the number of common bean root nodules and their effectiveness as compared to the control. The observed positive correlation between effective root nodules and common bean grain yield highlights the role of root nodulation in common bean yield. Overall, the locally produced microbial inoculant consortium demonstrated strong potential as a sustainable option to improve common bean yield, especially when integrated with chemical and organic fertilizers. These findings support sustainable agricultural practices and contribute to food security in resource-limited areas, despite limitations of one cropping season and geographic location that may not capture variability across other climates or soil types, and a lack of cost-benefit analysis to assess the economic effectiveness of different treatments used in this study. This highlights the need for further multi-season and multi-location trials, coupled with economic evaluations to support inoculant production and adoption by farmers.

## Supporting information

S1 Fig Assessment of common bean root nodule effectiveness based on nodule colouration showing two nodule types: non-senescent(NS-nodule/effective) and senescent (S-nodule/ non-effective) nodules [96]. (PDF)

S2 Table Summary Table Comparing Pre- and Post-Treatment Soil Properties. (DOCX)
