# Supplementary material for: Exploring the potential of microbial inoculant to enhance common bean (Phaseolus vulgaris L:) yield via increased root nodulation and soil macro-nutrients
Source: PLoS One. 2025 Oct 30;20(10):e0323854. doi: 10.1371/journal.pone.0323854 (PMC12574907; doi:10.1371/journal.pone.0323854)
Supplement: S1 Fig — (PDF) [file pone.0323854.s001.pdf]

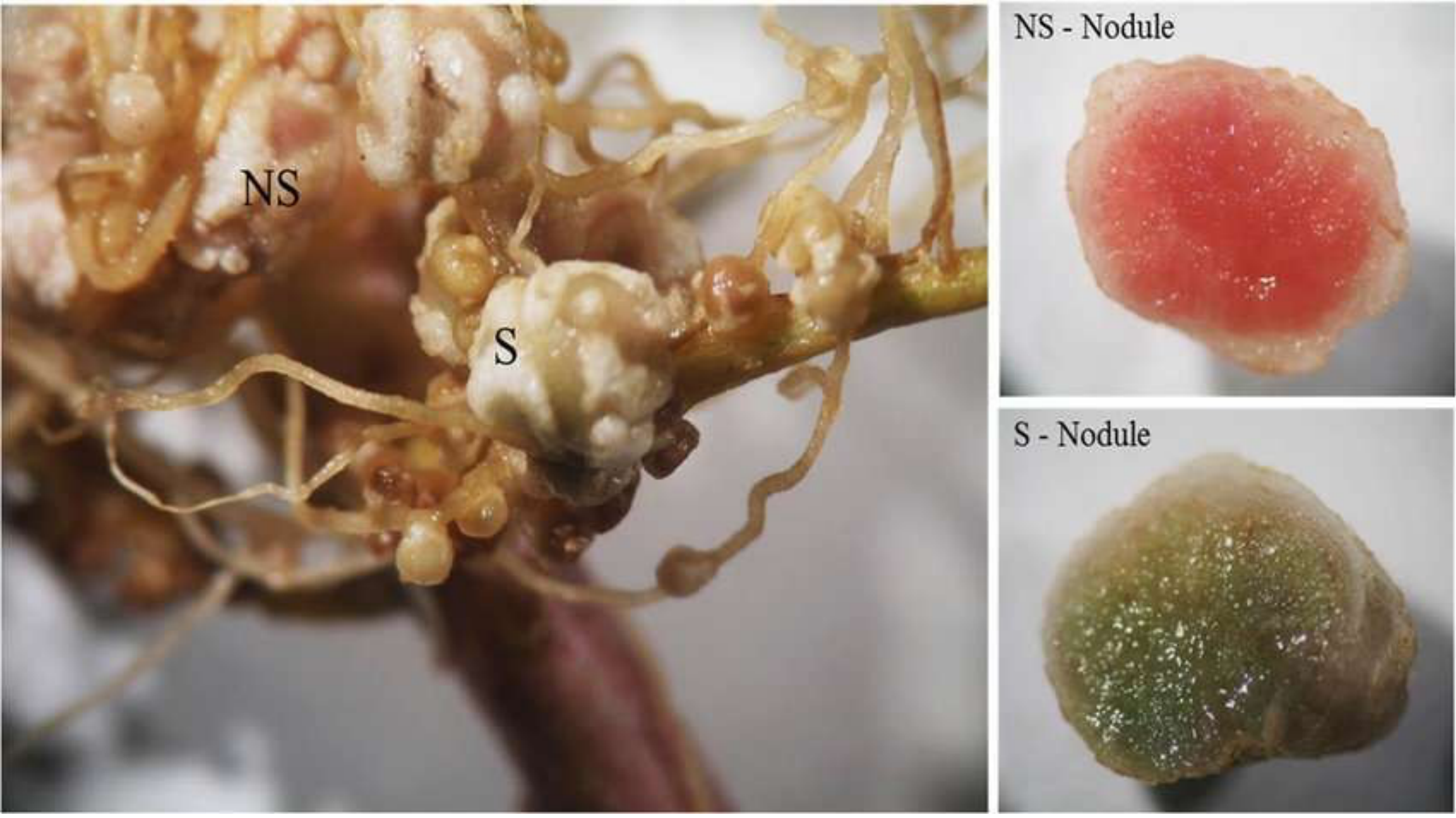

Reference

[96] da Silva HAP, Caetano VS, Pessoa DDV, Pacheco RS, Simoes-Araujo JS. Molecular and biochemical changes of aging-induced nodules senescence in common bean. Symbiosis. 2019;79, 33–48. https://doi.org/10.1007/s13199-019-00618-2
