# Supplementary material for: Exploring the potential of microbial inoculant to enhance common bean (Phaseolus vulgaris L:) yield via increased root nodulation and soil macro-nutrients
Source: PLoS One. 2025 Oct 30;20(10):e0323854. doi: 10.1371/journal.pone.0323854 (PMC12574907; doi:10.1371/journal.pone.0323854)
Supplement: S2 Table — (DOCX) [file pone.0323854.s002.docx]

**Summary Table Comparing Pre- and Post-Treatment Soil Properties**

| **Parameter** | **Pre-Treatment (Baseline Soil)** | **Post-Treatment**  **(Range Across Treatments)** | **Observations** |
| --- | --- | --- | --- |
| pH (H_2_O) | 6.60 ± 0.18 | 5.0 ± 0.17 (NPK) –  6.53 ± 0.12 (NPK+PM+MI) | Decreased under NPK; increased with microbes and integrated treatments. |
| Total Nitrogen (%) | 0.25 ± 0.02 | 0.12 ± 0.04 (NPK+PM) –  0.25 ± 0.01 (NPK+PM+MI) | Improved with microbial and integrated treatments; reduced in control/NPK. |
| Available P (mg/kg) | 8.02 ± 0.23 | 6.31 ± 0.16 (Control) –  8.68 ± 0.42 (NPK+PM+MI) | Highest in integrated; lowest in control. |
| Potassium (cmol/kg) | 1.62 ± 0.21 | 1.70 ± 0.22 (Control) –  2.80 ± 0.36 (NPK) | Increased notably under NPK and integrated treatments. |
| Calcium (cmol/kg) | 2.96 ± 0.30 | 2.04 ± 0.54 (Control) –  3.92 ± 0.39 (NPK) | Decreased in most treatments except NPK. |
| Magnesium (cmol/kg) | 1.24 ± 0.14 | 0.93 ± 0.12 (Control) –  2.20 ± 0.28 (NPK+PM) | Notably higher in integrated and microbe treatments. |

Control = no input; NPK = nitrogen, phosphorus and potassium; PM = poultry manure; MI = microbes
